# DKK2 promotes the progression of oral squamous cell carcinoma through the PI3K/AKT signaling pathway

**DOI:** 10.18632/aging.205864

**Published:** 2024-05-24

**Authors:** Wenbo Guo, Yun Qu, Yang Yu, Xueming Li, Zhuang Liang, Zhaoqi Wang, Tenglong Hu, Shan Zhou

**Affiliations:** 1 Department of Oral and Maxillofacial Surgery, The First Affiliated Hospital of Harbin Medical University, School of Stomatology, Harbin 150001, Heilongjiang, China; 2 Key Laboratory of Hepatosplenic Surgery, Ministry of Education, Harbin 150001, Heilongjiang, China; 3 Department of Orthodontics, The Second Affiliated Hospital of Harbin Medical University, Harbin 150001, Heilongjiang, China

**Keywords:** oral squamous cell carcinoma, DKK2, PI3K/AKT, hypoxia

## Abstract

Objective: This study aimed to investigate the impact of Dickkopf 2 (DKK2) on the progression of oral squamous cell carcinoma (OSCC) and explore its role in the PI3K/AKT signaling transduction pathway.

Materials and Methods: The study initially examined the expression of the DKK2 gene in OSCC tissues and normal tissues. Simultaneously, the expression of DKK2 in HOK cells and OSCC cells was verified, and changes in DKK2 expression under hypoxic conditions were detected. DKK2 overexpression and knockdown were performed in SCC-15 and CAL-27 cells. Subsequently, the effects of DKK2 on the proliferation, migration and invasion of OSCC were detected. Western blotting was employed to detect the expression of key proteins in the DKK2/PI3K/AKT signaling axis before and after transfection, and further explore the relevant molecular mechanisms.

Results: Compared to normal tissues, DKK2 expression was elevated in OSCC tissues. The expression of DKK2 in the SCC-15 and CAL-27 cell lines was higher than that in HOK cells, and hypoxic conditions could promote DKK2 expression. DKK2 overexpression promoted cell proliferation, migration, and invasion, while DKK2 knockdown inhibited these processes. DKK2 overexpression activated the PI3K/AKT pathway, while DKK2 knockdown suppressed this pathway.

Conclusion: This study suggests that hypoxic conditions enhance the expression of DKK2 in OSCC. DKK2 regulates the proliferation, migration, and invasion of OSCC through the PI3K/AKT signaling pathway.

## INTRODUCTION

Head and neck squamous cell carcinoma (HNSCC) accounts for approximately 1%–3% of all cancers globally, ranking as the sixth most common cancer worldwide [1]. Malignant tumors originating from squamous cells contribute significantly to oral cancer, comprising over 90% of cases [2]. HNSCC is the most prevalent malignant tumor in the head and neck region, and its incidence has been gradually increasing in recent years [3]. HNSCC is a burden faced globally, with a high mortality rate of approximately 500,000 deaths in 2022 due to its characteristics of late diagnosis, rapid local invasion, high metastasis, and difficult management [4]. Despite improvements in economic conditions and living standards, the mortality rate of HNSCC continues to rise. Survival rates for cancer are based on its prognostic indicators, such as grade, stage, metastasis, and invasion, and despite treatments such as surgery, chemotherapy, and radiation, survival rates for patients with HNSCC remain at only about 50 percent [5]. OSCC has the highest incidence of HNSCC and a high mortality rate [6]. This is attributed to the poor prognosis of oral squamous cell carcinoma (OSCC) [7], with over 50% of diagnosed cases posing a threat to life [8]. Therefore, it is crucial to propose new treatment strategies for OSCC patients and explore the relevant mechanisms underlying the progression of OSCC to improve the patient’s outcomes.

The WNT signaling pathway plays a crucial role in the development of human cancers [9]. Studies have demonstrated an association between dysregulation of WNT signaling and the occurrence of OSCC [10]. Additionally, epigenetic modifications of the Dickkopf family (DKK1-4) have been shown to play a vital role in regulating WNT signal transduction [11]. Research indicates that Dickkopf 2 (DKK2) acts as an oncogene, promoting proliferation, migration, and invasion of cells in OSCC [12]. DKK2 may function as an inhibitor or activator of the Wnt/β-catenin pathway, depending on the cellular context [13]. For instance, DKK2 has been reported to promote the Wnt/β-catenin pathway, enhancing proliferation and invasion of prostate cancer cells [11].

Dickkopf 2 (DKK2) has been identified as a key regulatory factor in various cancers. In pancreatic ductal adenocarcinoma, upregulation of DKK2 indicates poor prognosis [14], while in gastric cancer, DKK2 acts as a tumor suppressor gene [15]. A study by Japanese scholar Akiko Kawakita reported that MicroRNA-21 promotes oral cancer invasion through the Wnt/β-Catenin pathway by targeting DKK2. The study found that miRNA-21 promotes oral cancer invasion by downregulating the Wnt antagonist gene DKK2 [12].

Due to the complexity of the development of OSCC, we hypothesize that DKK2 may still influence the progression of OSCC through other molecular regulatory mechanisms. Therefore, this study aims to validate the role of DKK2 in the progression of OSCC and preliminarily explore the downstream molecular mechanisms of DKK2. Our findings confirm that DKK2 plays a promoting role in the PI3K/AKT pathway in OSCC and that its expression is promoted in a hypoxic microenvironment.

## MATERIALS AND METHODS

### Sample collection

Tissue samples were collected from patients undergoing OSCC resection surgery at the Department of Oral and Maxillofacial Surgery, Affiliated Stomatology Hospital, Harbin Medical University. Both OSCC tissue samples and adjacent non-cancerous tissue samples were obtained.

### Cell culture

HOK and CAL27 cells were cultured in Dulbecco’s modified Eagle medium supplemented with 10% fetal bovine serum. SCC15 cells were cultured in a 1:1 mixture of Dulbecco’s modified Eagle’s medium and Ham’s F12 medium supplemented with 400 ng/ml hydrocortisone and 10% fetal bovine serum. Normoxic conditions were at 37°C, 95% air and 5% CO_2_. Hypoxic conditions were at 37°C, 5% CO_2_, 1% O_2_, nitrogen balance.

### Cell transfection

SiRNA and overexpression plasmids used in this experiment were synthesized by Guangzhou Ruibo Biological Co., Ltd., China. Transfection reagents included Polyplus transfection and Lipofectamine 2000 (Thermo Fisher, # 11668027). Cells in logarithmic growth phase were seeded in six-well plates, with an appropriate cell density per well. After cells reached approximately 70% confluence, siRNA or overexpression plasmids were added to the corresponding groups, and after 8 hours, the medium was replaced with normal culture medium. RNA and protein were collected 24–48 hours later to validate the efficiency of knockdown and overexpression.

### EdU proliferation assay

Cells in logarithmic growth phase were seeded into six-well plates according to different groups. The BeyoClick™ EdU-488 Cell Proliferation Assay Kit (Product Number: C0071S) was used for EdU labeling, cell fixation, washing, permeabilization, EdU detection, and nuclear staining. Randomly selecting and capturing six fields under a fluorescence microscope, cells were observed and counted. The percentage of cells in the proliferative phase was calculated as (green fluorescence count/blue fluorescence count) × 100%.

### Cell scratch assay

Cells in logarithmic growth phase were seeded into six-well plates and allowed to reach over 90% confluence. A straight line was scratched using a 200 μL pipette tip, followed by PBS washing, and 2 mL serum-free culture medium was added to each well. Photographs were taken under a microscope at 0 hours (scratch time) and 24 hours. The scratch width was recorded, and the cell migration area was calculated using ImageJ 1.8.0. Cell migration rate (%) = (migration area/initial area) × 100%.

### Migration assay

Matrigel was coated on the bottom of the upper chamber. SCC15 and CAL27 cells in logarithmic growth phase were prepared as serum-free cell suspensions and added to the upper chamber, with a cell density of approximately 4 × 10^5^ cells/mL. In the lower chamber, 400 μL DMEM culture medium containing 10% serum was added. After 72 hours of incubation, non-migratory cells were removed, and the filter membrane was fixed, stained, and photographed. Nine random fields were observed and counted.

### Quantitative real-time PCR

Cells were digested and collected in EP tubes. Total RNA was extracted using the SevenFast Total RNA Extraction Kit for Cells (Product Number: SM130-01). Extracted RNA was reverse transcribed into cDNA using the All-in-one First Strand cDNA Synthesis Kit II (Product Number: SM134-01) according to the manufacturer's instructions. Real-time quantitative PCR was performed using 2× SYBR Green qPCR MasterMix II (Product Number: SM143-01). The primer sequences used in this study are as follows: GAPDH forward primer: 5′-GTCTCCTCTGACTTCAACAGCG-3′, GAPDH reverse primer: 5′-ACCACCCTGTTGCTGTAGCCAA-3′, DKK2 forward primer: 5′-GGATGGCAGAATCTAGGAAGACC-3′, DKK2 reverse primer: 5′-CTGATGGAGCACTGGTTTGCAG-3′.

### Western blot

After removing the cell culture medium, RIPA buffer (containing 1× protease inhibitor and 1× phosphatase inhibitor) was added to lyse cells on ice. The lysates were centrifuged at 12000 × g for 15 minutes, and the protein supernatant was collected and quantified using the BCA Protein Concentration Assay Kit (Beyotime, Product Number: P0012S). Proteins were separated by SDS-PAGE, transferred to NC membranes, and blocked with 5% skim milk for 2 hours. The membranes were then incubated with DKK2 Rabbit pAb (A14874) at 4°C overnight. After incubation with secondary antibodies, bands were visualized using the Odyssey dual-color infrared fluorescence imaging system, and band intensities were quantified. Relative protein expression level = (band intensity of the target band/band intensity of the reference band) × 100%.

### Gene co-expression analysis

TCGA-HNSC (Head and Neck Squamous Cell Carcinoma) project data were downloaded and curated from the TCGA database (https://portal.gdc.cancer.gov/). OSCC-related RNAseq data in TPM format were extracted, and co-expression analysis of DKK2 and other genes was performed. Samples were divided into high DKK2 expression, low DKK2 expression groups based on the median expression, and the results were visualized using the ggplot2 package.

### Enrichment analysis

To explore the functional relevance of DKK2 and its co-expressed genes, functional and pathway enrichment analyses were conducted. Gene Ontology (GO) functional annotation analysis, including biological processes (BP), molecular function (MF), and cellular component (CC), was performed using the R package clusterProfiler. Additionally, the cBioPortal database (https://www.cbioportal.org/) was utilized to analyze pathways affected by DKK2.

### Statistical analysis

All results are presented as the mean ± standard deviation. Statistical analysis of differences between samples was performed using two-tailed Student’s *t*-tests, and *p* < 0.05 was defined as significant.

## RESULTS

### DKK2 is highly expressed in OSCC

**Figure 1:**
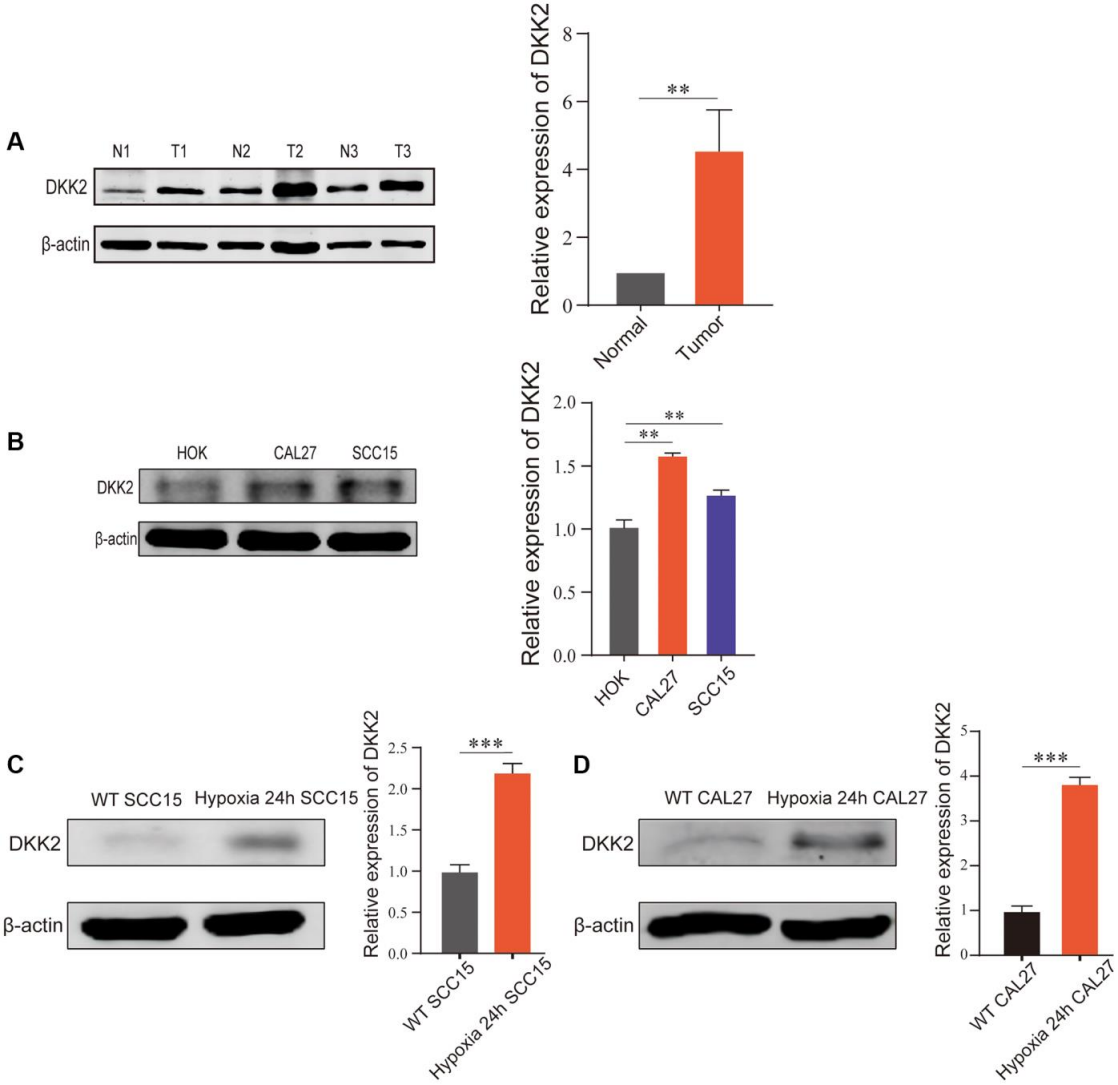
**DKK2 expression differences in oral squamous cell carcinoma.** (**A**) DKK2 protein expression in human oral squamous cell carcinoma tissues (T) and normal oral mucosa tissues (N). (**B**) DKK2 protein expression in HOK, CAL27, and SCC15. (**C**) DKK2 expression in hypoxic and normoxic in SCC15 cell. (**D**) DKK2 expression in hypoxic and normoxic in CAL27 cell. ^*^p < 0.05; ^**^p < 0.01; ^***^p < 0.001; ^****^p < 0.0001.

Firstly, we examined the expression of DKK2 in OSCC tissues and adjacent non-cancerous tissues using Western Blot. The results showed a significant upregulation of DKK2 in OSCC tissues compared to adjacent non-cancerous tissues (Figure 1A). Subsequently, we validated DKK2 expression in OSCC cell lines. Western Blot analysis of normal oral mucosal cell line HOK and OSCC cell lines CAL27 and SCC15 revealed a significant overexpression of DKK2 in both OSCC cell lines (Figure 1B). As OSCC is a solid tumor with a characteristic hypoxic center, which may have a crucial impact on tumor development, we simulated hypoxic conditions *in vitro* to investigate the changes in DKK2 expression in CAL27 and SCC15 cell lines. The results showed an increased expression of DKK2 under hypoxic conditions compared to normoxic conditions in both cell lines (Figure 1C, 1D).

### DKK2 promotes the progression of oral squamous cell carcinoma

To further explore the biological functions of DKK2 in the development of OSCC, we manipulated DKK2 expression in OSCC cell lines. Due to the relatively low expression of DKK2 in SCC15, we chose to overexpress DKK2 in SCC15 and knock down DKK2 in CAL27.

**Figure 2:**
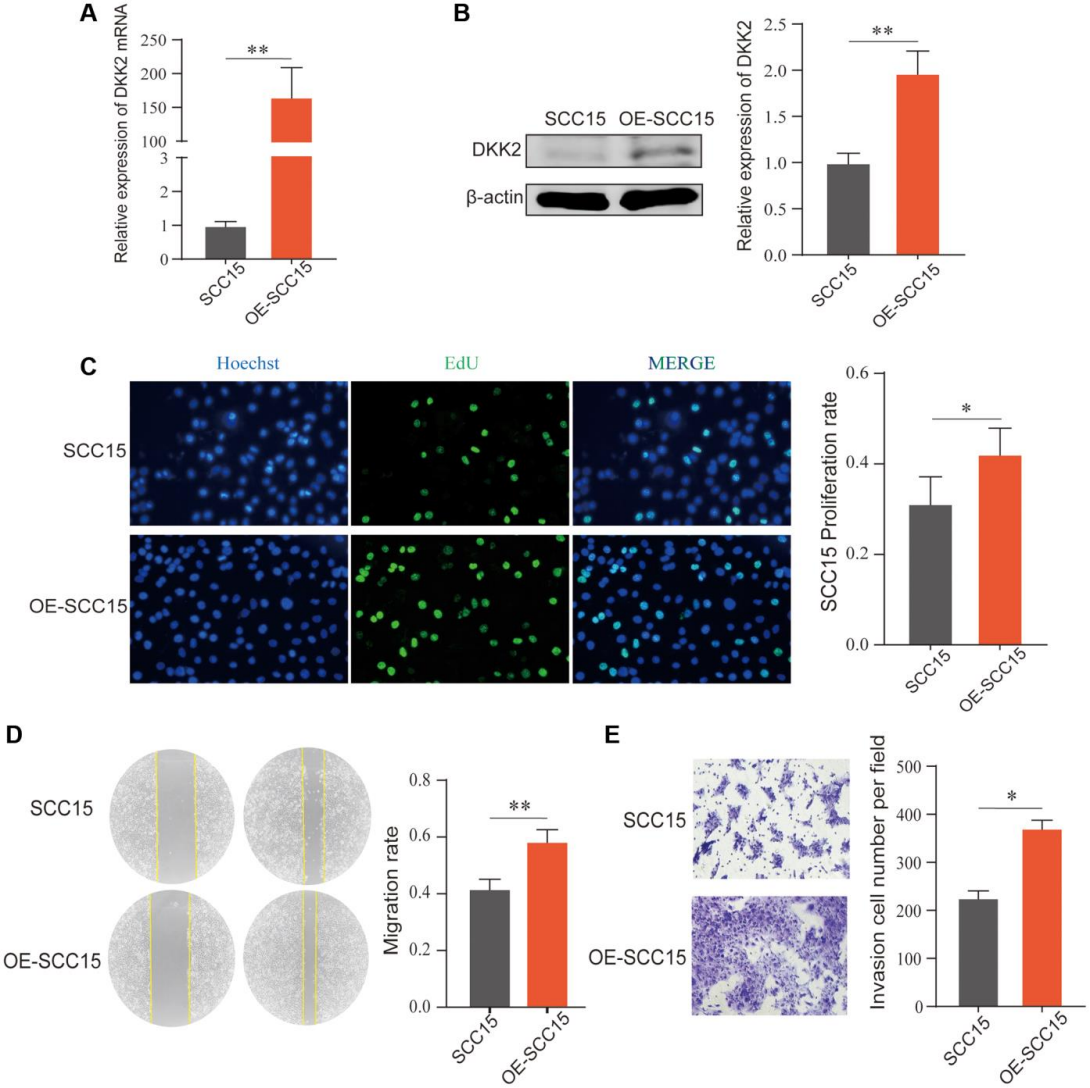
**Overexpression of DKK2 and the progression of oral squamous cell carcinoma.** (**A**) Reverse transcription-quantitative polymerase chain reaction was used to verify the overexpression efficiency of the SCC15 cell line. (**B**) Western blot was used to detect the overexpression efficiency of SCC15 cell line. (**C**) After overexpression of DKK2, the proliferation of transfected SCC15 cell line was detected using EdU assay. (**D**) After overexpression of DKK2, the migration of cells was detected using scratch assay. (**E**) After overexpression of DKK2, the invasion of cells was detected using Transwell assay. ^*^p < 0.05; ^**^p < 0.01; ^***^p < 0.001; ^****^p < 0.0001.

After transfection of the DKK2 overexpression plasmid in SCC15 cells, we confirmed the overexpression efficiency using qPCR and Western Blot. The results showed a significant increase in DKK2 mRNA and protein compared to the empty vector control group (Figure 2A, 2B). Subsequent biological function experiments revealed that overexpression of DKK2 significantly enhanced the proliferation ability of OSCC cells, as indicated by the EdU assay (Figure 2C). Furthermore, scratch healing experiments demonstrated that overexpression of DKK2 increased the migration ability of SCC15 cells (Figure 2D), and Transwell assays showed a significant enhancement in the invasive capability of OSCC cells with DKK2 overexpression (Figure 2E).

**Figure 3:**
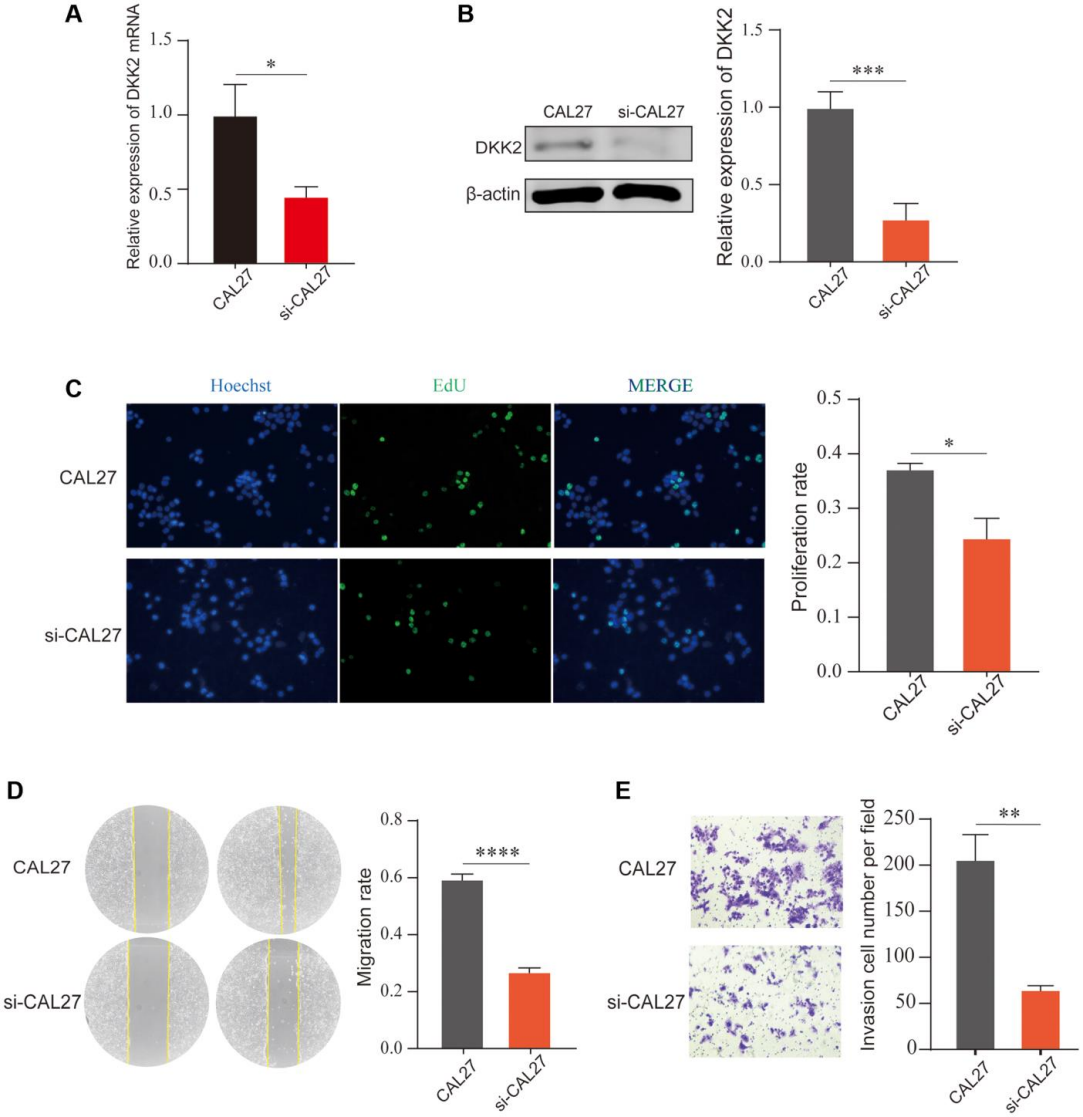
**Knockdown of DKK2 can reduce the progression of oral squamous cell carcinoma.** (**A**) Reverse transcription-quantitative polymerase chain reaction was used to verify the knockdown efficiency of CAL27 cell line. (**B**) Western blot was used to detect the knockdown efficiency of CAL27 cell line. (**C**) After knockdown of DKK2, EdU assay was used to detect the proliferation of transfected CAL27 cell line. (**D**) After knockdown of DKK2, scratch assay was used to detect the migration of cells. (**E**) After knockdown of DKK2, Transwell assay was used to detect the invasion of cells. ^*^p < 0.05; ^**^p < 0.01; ^***^p < 0.001; ^****^p < 0.0001.

Similarly, we knocked down DKK2 in another OSCC cell line, CAL27, and confirmed the significant knockdown efficiency using qPCR and Western Blot (Figure 3A, 3B). EdU assay results showed that the proliferation ability of OSCC cells decreased after DKK2 knockdown (Figure 3C). Scratch healing and Transwell assay results also indicated a reduction in the malignant biological behavior of OSCC cells, with decreased migration and invasion abilities after DKK2 knockdown (Figure 3D, 3E). Our experiments suggest that DKK2 plays a strong promoting role in the progression of OSCC, increasing the malignant biological behavior of OSCC cells and potentially serving as an effective therapeutic target.

### Co-expression heatmap of DKK2 downstream genes

**Figure 4:**
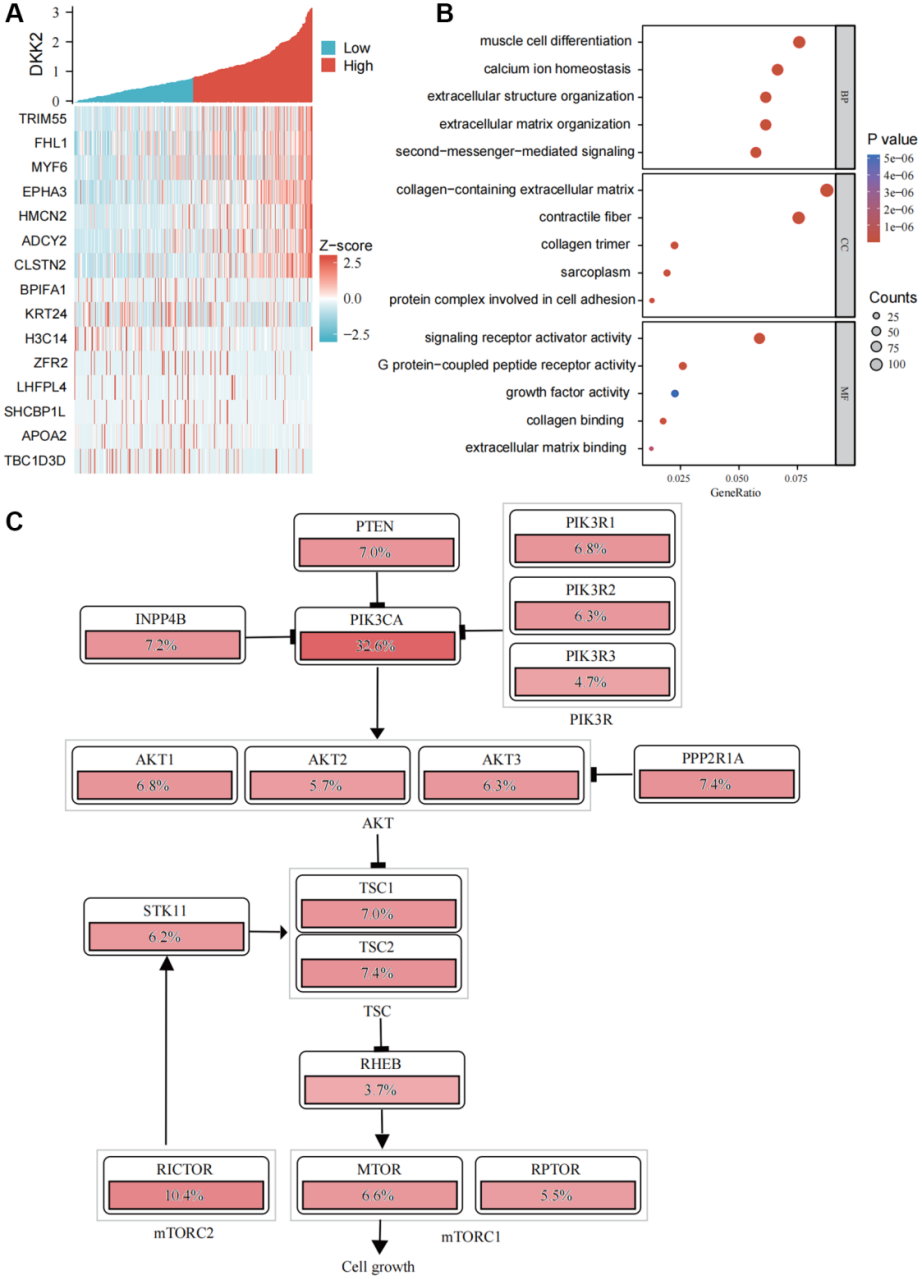
**Functional enrichment analysis of DKK2.** (**A**) Heatmap of DKK2 co-expressed genes in oral squamous cell carcinoma. (**B**) Gene Ontology enrichment analysis. (**C**) Schematic diagram of DKK2 affecting the PI3K/AKT signaling pathway. ^*^p < 0.05; ^**^p < 0.01; ^***^p < 0.001; ^****^p < 0.0001.

We used bioinformatics analysis to generate a co-expression heatmap of potential downstream genes of DKK2 (Figure 4A) and performed functional enrichment to explore the possible mechanisms by which DKK2 functions in OSCC (Figure 4B). The results indicated that DKK2 and its downstream genes might affect the extracellular matrix, consistent with our previous findings on the impact of DKK2 on the migration and invasion capabilities of OSCC. Mechanistically, DKK2 may influence second messenger signaling and receptor activation. Pathway analysis also suggested that, in addition to its impact on the Wnt signaling pathway, DKK2 has a significant regulatory role in the PI3K/AKT signaling pathway (Figure 4C).

### DKK2 promotes the progression of OSCC through the PI3K/AKT signaling pathway

**Figure 5:**
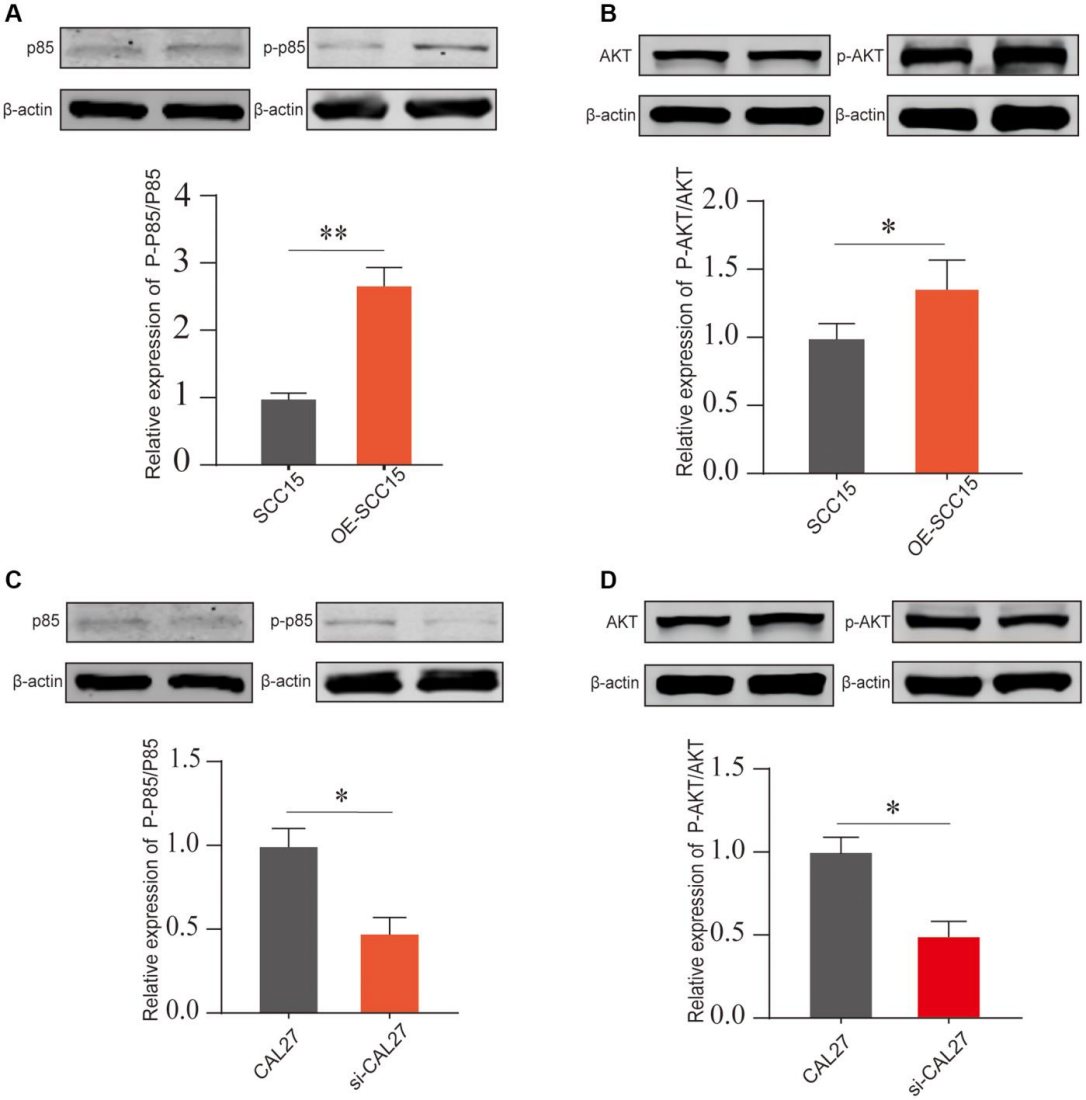
**The PI3K/AKT pathway activation after DKK2 knockdown/overexpression.** (**A**, **B**) After overexpressing DKK2 in SCC15 cells, the activation of the PI3K/AKT pathway was detected by Western blot assay. (**C**, **D**) After knocking down DKK2 in CAL27 cells, the activation of the PI3K/AKT pathway was detected by Western blot assay. ^*^p < 0.05; ^**^p < 0.01; ^***^p < 0.001; ^****^p < 0.0001.

We next molecularly validated the impact of DKK2 on the PI3K/AKT signaling pathway. In SCC15, overexpression of DKK2 led to a significant increase in phosphorylation of p85 and, while total protein expression remained nearly unchanged (Figure 5A). Similarly, DKK2 also increased the phosphorylation level of AKT (Figure 5B). This indicates that increased expression of DKK2 can promote the activation of the PI3K/AKT signaling pathway, thereby enhancing the proliferation of OSCC cells. We also conducted the same validation in CAL27 cells; after knocking down DKK2, a decrease in the phosphorylation levels of p85 and AKT proteins was observed (Figure 5C, 5D). This suggests that the high expression of DKK2 in OSCC can promote the activation of the PI3K/AKT signaling pathway, contributing to the progression of OSCC.

## DISCUSSION

In this study, we showed that DKK2 showed high expression in cancer tissues and in OSCC cell lines. DKK2 affects the proliferation, migration and invasion of OSCC through the PI3K/AKT signaling pathway. And hypoxic conditions can promote the expression of DKK2 in OSCC. Regulation of DKK2 expression may become a new idea in the treatment of OSCC.

DKK2 is a secreted protein, and extensive research has indicated its involvement in tumor cell proliferation, survival, migration, and invasion. In renal carcinoma, the expression of DKK2 is epigenetically suppressed, and its ectopic expression reduces invasion and induces apoptosis of cancer cells [16]. DKK2 also increases tumor growth and metastasis through the transcriptional upregulation of matrix metalloprotease-1 in Ewing’s Sarcoma [17–20]. Additionally, high expression of DKK2 has also been reported in colorectal cancer [21–24]. In oral cancer, Akiko Kawakita and colleagues found that miR-21 promotes oral cancer invasion by down-regulating the Wnt antagonist gene DKK2 [7]. Sedigheh Kheirandish and colleagues discovered that hypomethylation of DKK2 in higher grades of tumors versus semi-methylation pattern in low grades, it may indicate that overexpression of the DKK2 gene is necessary for the tumor transition from low to high grades [25]. These findings suggest that DKK2 plays either oncogenic or tumor-suppressive roles in different cell types or environments.

One of the characteristics of solid malignant tumors is the relative hypoxia within the tumor mass [26, 27]. This is because rapidly growing malignant tumors disrupt the balance between oxygen consumption and supply, leading to a hypoxic environment inside cancer cells. The hypoxic environment of cancer cells activates a series of cascading factors, and the interaction of these factors contributes to issues such as cancer metastasis, invasion, resistance changes, and poor prognosis [28–31]. Therefore, we examined the changes in DKK2 under simulated *in vivo* hypoxic conditions and confirmed that the expression of DKK2 increases under hypoxic conditions. This is consistent with the results of Yu Zhao and colleagues in their study on RPE cells under hypoxic conditions [32].

However, the pathogenesis of tumor hypoxia is multifactorial, with both acute and chronic factors influencing it [33, 34]. Therefore, the ability of purely induced hypoxic conditions to accurately simulate the *in vivo* microenvironment state remains to be discussed. The spatial distribution of hypoxia within tumors is usually significantly heterogeneous, often changing over time, and significant changes in oxygenation status may even occur between several cell layers [35–37]. Therefore, the focus of future work may lie in endogenous markers of hypoxia within tumors, using these markers to select more pure hypoxic cells for further experiments. At the same time, we hope that animal experiments can be supplemented in the future. But in what way to provide tumor cells with a hypoxic microenvironment is something to think about.
